# Bioactivity and Cell Compatibility of β-Wollastonite Derived from Rice Husk Ash and Limestone

**DOI:** 10.3390/ma10101188

**Published:** 2017-10-17

**Authors:** Roslinda Shamsudin, Farah ‘Atiqah Abdul Azam, Muhammad Azmi Abdul Hamid, Hamisah Ismail

**Affiliations:** School of Applied Physics, Faculty of Science & Technology, Universiti Kebangsaan Malaysia, Bangi 43600, Selangor, Malaysia; linda@ukm.edu.my (R.S.); qfaara@gmail.com (F.A.A.A.); azmi@ukm.edu.my (M.A.A.H.)

**Keywords:** autoclaving, bioactive, wollastonite, green synthesize, rice husk ash

## Abstract

The aim of this study was to prepare β-wollastonite using a green synthesis method (autoclaving technique) without organic solvents and to study its bioactivity. To prepare β-wollastonite, the precursor ratio of CaO:SiO_2_ was set at 55:45. This mixture was autoclaved for 8 h and later sintered at 950 °C for 2 h. The chemical composition of the precursors was studied using X-ray fluorescence (XRF), in which rice husk ash consists of 89.5 wt % of SiO_2_ in a cristobalite phase and calcined limestone contains 97.2 wt % of CaO. The X-ray diffraction (XRD) patterns after sintering showed that only β-wollastonite was detected as the single phase. To study its bioactivity and degradation properties, β-wollastonite samples were immersed in simulated body fluid (SBF) for various periods of time. Throughout the soaking period, the molar ratio of Ca/P obtained was in the range of 1.19 to 2.24, and the phase detected was amorphous calcium phosphate, which was confirmed by scanning electron microscope with energy dispersive X-ray analysis (SEM/EDX) and XRD. Fourier-transform infrared spectroscopy (FTIR) analysis indicated that the peaks of the calcium and phosphate ions increased when an amorphous calcium phosphate layer was formed on the surface of the β-wollastonite sample. A cell viability and proliferation assay test was performed on the rice husk ash, calcined limestone, and β-wollastonite samples by scanning electron microscope. For heavy metal element evaluation, a metal panel that included As, Cd, Pb, and Hg was selected, and both precursor and β-wollastonite fulfilled the requirement of an American Society for Testing and Materials (ASTM F1538-03) standard specification. Apart from that, a degradation test showed that the loss of mass increased incrementally as a function of soaking period. These results showed that the β-wollastonite materials produced from rice husk ash and limestone possessed good bioactivity, offering potential for biomedical applications.

## 1. Introduction

Wollastonite is a naturally occurring mineral that consists of calcium metasilicate with the chemical formula CaSiO_3_. Wollastonite exists in two primary mineral phases; β-wollastonite (wollastonite), which exists at low temperatures, and α-wollastonite or pseudowollastonite at high temperatures [1,2,3]. Wollastonite is generally used in dental materials and biomaterials [4,5,6,7,8,9], concrete [10], porcelain [11,12], heat insulating ceramics [13], and cement [14] due to its various valuable properties such as good biocompatibility, fluxing characteristics, low shrinkage, and good strength.

Previous studies have successfully synthesized wollastonite powders using chemicals such as calcium nitrate and fumed silica as the precursors [15]. Meanwhile, there are also studies that purchased wollastonite from chemical suppliers [16]. This research offers an alternative method, diversifying the use of green synthesizing from local resources such as agricultural waste and mineral resources. In the present study, rice husk ash (RHA) and local limestone were used to produce β-wollastonite in order to maximize the utilization of waste and local sources. The starting material is an agricultural waste from rice husks, which is burned to obtain the optimum silica content. At 900 °C, rice husk ash has a higher silica content of about 97 wt % that of other agricultural wastes such as palm oil ash [17] and wheat straw ash [18]. Thus, rice husk ash is the preferred silica precursor compared to silica sand for producing wollastonite [2,19]. Moreover, the use of rice husk can help to reduce open burning practices, particularly in Malaysia, while controlling pollution problems such as haze and global warming [20]. Meanwhile, calcium oxide was obtained from limestone, a mineral that is easily available in Malaysia.

Reports from previous studies have shown that wollastonite is highly bioactive and a suitable material for implants [1,5]. Thus, this study seeks to investigate the characteristics and nature of bioactive β-wollastonite from RHA, that is, to determine the cell attachment via a cell viability and proliferation assay test, the heavy metal element content, the formation behaviour of the apatite layer, the degradation of the β-wollastonite sample after soaking in simulated body fluid (SBF), and its potential as an implantable material.

## 2. Results and Discussion

### 2.1. Characteristics of Rice Husk Ash (RHA), Limestone and β-Wollastonite Powder

The results of the elemental analyses for the rice husk ash (RHA) and calcined limestone using an X-ray fluorescence (XRF) are shown in Table 1. The silica content of the RHA was 89.5 wt %, which was similar than that reported by Jenkins et al. [21]. There were other impurities such as K_2_O, P_2_O_5_, MgO, Al_2_O_3_, CaO, and others. The main reason for selecting the RHA was its silica content, which is the highest compared to other agricultural wastes [17,18]. Rice husk is also easily obtained from paddy fields. Calcined limestone contained about 97.22 wt % calcium oxide, and the remaining elements were MgO and others. The RHA has a mean particle size of 20.82 μm and density of 2.50 g·cm^−3^. The theoretical density of silica, which is 2.65 g·cm^−3^, was used as a comparison to RHA as the main content of the ash is silica [19]. For calcined limestone, the mean particle size is 6.90 μm and the density is 3.02 g·cm^−3^, which is close to the typical density of 3.35 g·cm^−3^ [22].

**Figure 1:**
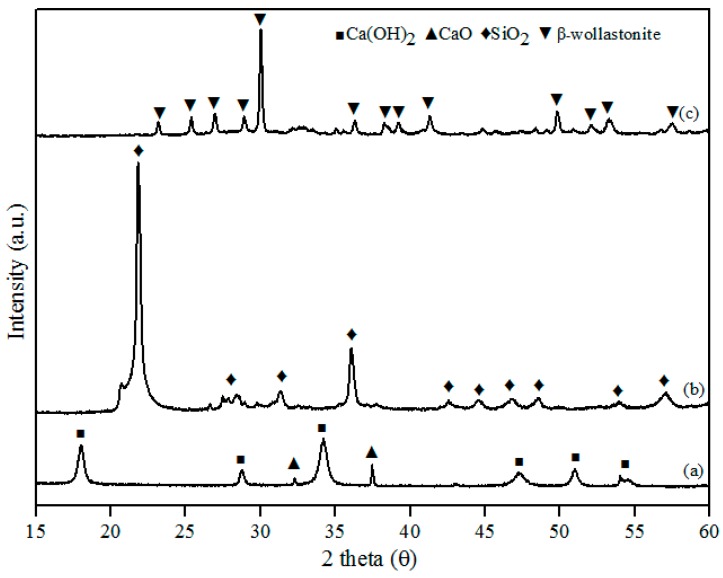
X-ray diffraction (XRD) patterns for (**a**) calcined limestone; (**b**) rice husk ash; and (**c**) β-wollastonite.

This study successfully developed β-wollastonite from a combination of RHA derived silica (SiO_2_) and limestone derived calcium oxide (CaO). The CaO:SiO_2_ ratio and the sintering temperature were selected based on the phase diagram of the CaO-SiO_2_ system [23] and were 55:45 and 2 h, respectively. The particle size and density results for the obtained β-wollastonite are 28.12 µm and 3.03 g·cm^−3^, respectively. The increase in particle size was due to the coarsening mechanism of silicon from RHA during the sintering process [24], whilst the density is close to its theoretical density of 2.86 to 3.09 g·cm^−3^ [19]. The phase-formation behaviour of β-wollastonite during the sintering process was investigated using XRD, as shown in Figure 1. Calcined limestone (Figure 1a), CaO (International Centre for Diffraction Data, ICDD number: 00-37-1497), and Ca(OH)_2_ (ICDD number: 00-44-1481) peaks were also observed as it is difficult for calcium oxide to remain in its original phase due to its moisture-absorbing property. Calcium oxide may also revert to its original phase, CaCO_3_ [25]. The peaks observed at 32.2 and 37.4 (degrees) correspond to the (220) and (200) directions, respectively, for CaO, whereas the peaks observed at 18.0, 28.8, 34.0, 47.1, 50.9, and 54.4 (degrees) corresponded to the (001), (100), (101), (102), (110), and (111) directions, respectively, for Ca(OH)_2_. The cristobalite phase (ICDD number: 00-82-0512) was found to exist in the RHA following firing at 950 °C (Figure 1b). These peaks (2 theta) of RHA were observed at 22.0, 31.2, 36.2, 42.7, 44.5, 46.8, 48.6, and 57.1 (degrees) which correspond to the (101), (102), (200), (211), (202), (113), (212), and (301) directions, respectively. While in Figure 1c, the peaks observed at 23.1, 25.4, 26.9, 28.9, 30.0, 35.5, 36.2, 38.2, 39.2, 41.3, 50.9, 52.0, and 53.2 corresponded to the (400), (002), (−202), (202), (−320), (402), (412), (−203), (−422), (040), (004), (241), and (523) planes, respectively; this is the β-wollastonite phase (ICDD number: 00-043-1460). The β-wollastonite phase was obtained after 8 h autoclaving and 2 h sintering reaction between cristobalite and calcined limestone, as shown in the XRD results (Figure 1c). As no other phases were detected, it shows that a complete reaction between cristobalite and calcined limestone had occurred, which produced single-phase β-wollastonite.

Table 2 shows the impurities of the RHA, calcined limestone, and β-wollastonite, indicating that heavy metals such as arsenic (As), cadmium (Cd), lead (Pb), and mercury (Hg) satisfied the requirements to be used as a biomaterial with reference to the standard specifications ASTM F1538-03 [26]. No mercury (Hg) was detected in the RHA, the calcined limestone, or the obtained β-wollastonite. Thus, it was established that the contents of RHA, calcined limestone, and β-wollastonite were secure for use in the human body as implants.

**Figure 2:**
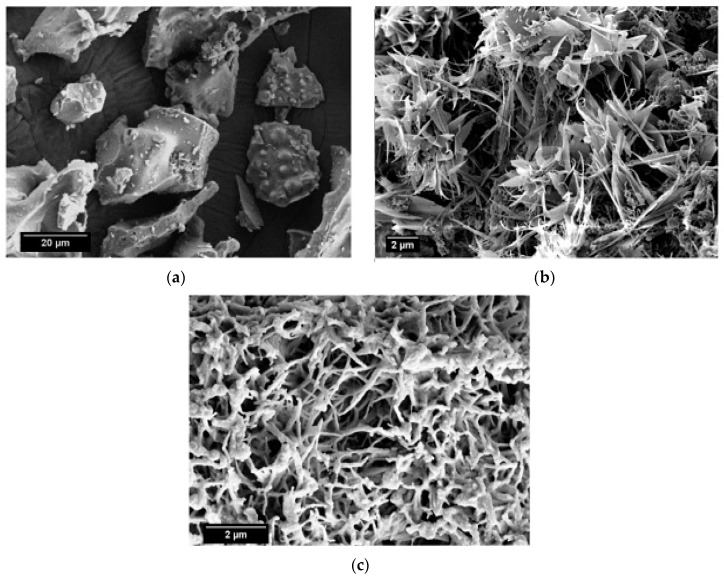
SEM micrographs of (**a**) rice husk ash; (**b**) calcined limestone; and (**c**) β-wollastonite.

SEM micrographs of the calcined limestone at 1100 °C, as well as RHA and β-wollastonite sintered at 950 °C, are shown in Figure 2. Based on these micrographs, it can be seen that the RHA is of an irregular shape (Figure 2a), while the calcined limestone shows an irregular shape with a flaky structure (Figure 2b). However, after the sintering process, the β-wollastonite exhibited a dendrite-like structure (Figure 2c). It was expected that these samples would contain only β-wollastonite because the sintering temperature for β-wollastonite is from 870 to 1100 °C [23]. This result was definitely confirmed by the presence of intense peaks related to β-wollastonite.

**Figure 3:**
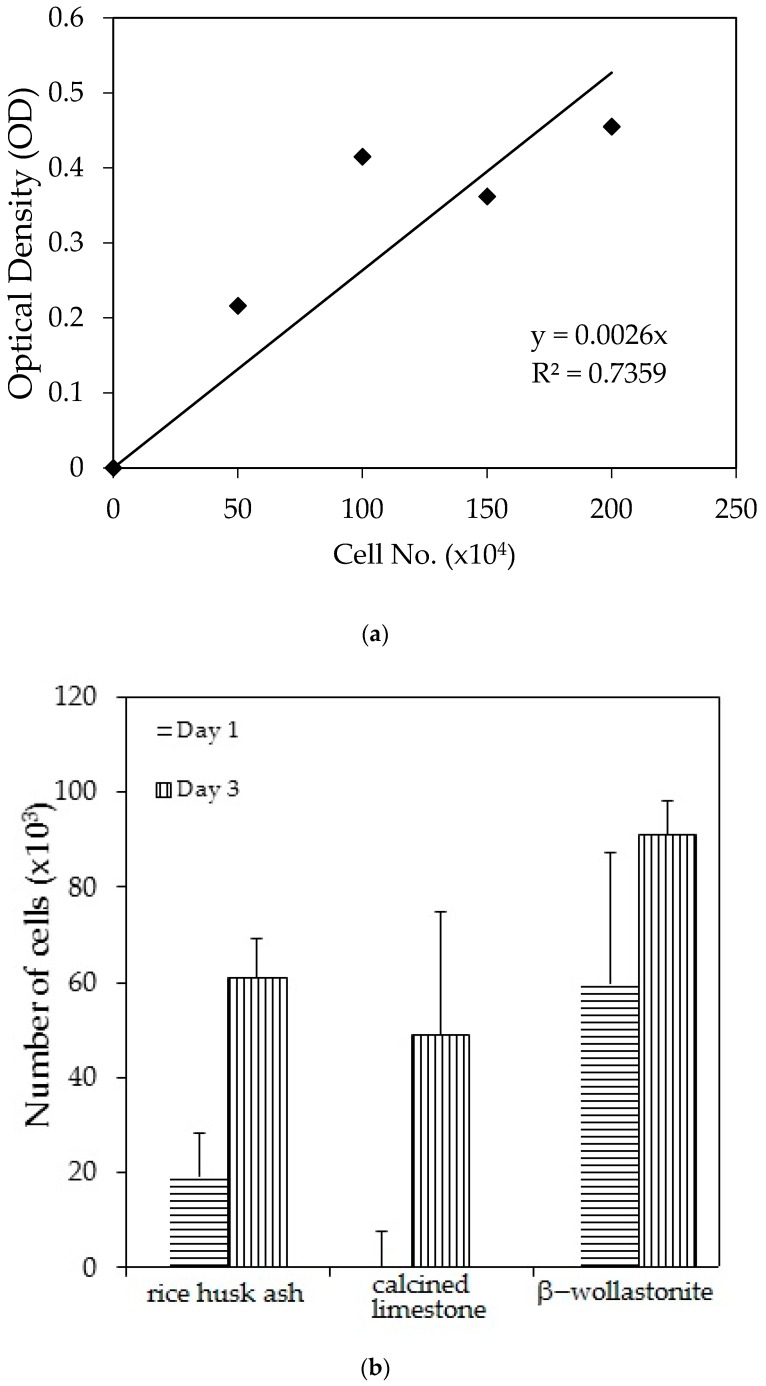

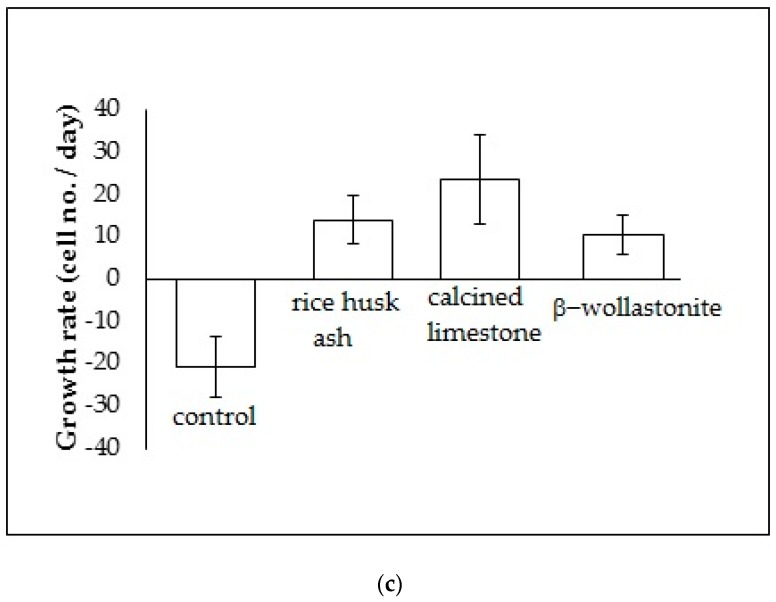
Cell viability test analyses show the; (**a**) reference curve for cell density on the culture dish; (**b**) cell distribution across different material discs; and (**c**) cell growth rates across the different material discs of rice husk ash, calcined limestone, and β-wollastonite powder samples for cell viability test.

The cell viability test result shows that the distribution of cells increased with increased soaking (Figure 3b); refer to the reference curve for the cell density on the culture dish (Figure 3a). It was found that the distribution of cells on the β-wollastonite is the highest compared to that on RHA and calcined limestone for days 1 and 3. The numbers of distribution cells on day 3 for the RHA, calcined limestone, and β-wollastonite were 61.00, 49.00, and 91.00 (×10^3^), respectively. The growth rate of the cells for the RHA, calcined limestone, and β-wollastonite samples are shown in Figure 3c. Calcined limestone shows the highest cell growth rate compared to RHA and β-wollastonite, which each exhibited growth rates of 25, 15, and 10 (×10^3^) cell No./day, respectively. This is due to the natural property of the calcined limestone mineral that promotes the cell seeding process. All the starting materials are biocompatible, which is related to the cell seeding process at the surface of RHA and calcined limestone. Therefore it is shown that the β-wollastonite samples also have good biocompatibility properties in instances when a cell seeding process is allowed on its surface. Among the factors that affect the seeded cells in this study is the sample surface’s roughness; a rough surface is greatly favored by the cells compared to a smoother surface [27]. In addition, cell adhesion and growth greatly depend on the chemical composition and topography of the surface sample [28]. Thus the RHA, calcined limestone, and β-wollastonite samples used in this study are nontoxic to human osteoblast cells, where the cells are able to seed on the surface of all the samples.

### 2.2. Characteristics of the Soaked β-Wollastonite

**Figure 4:**
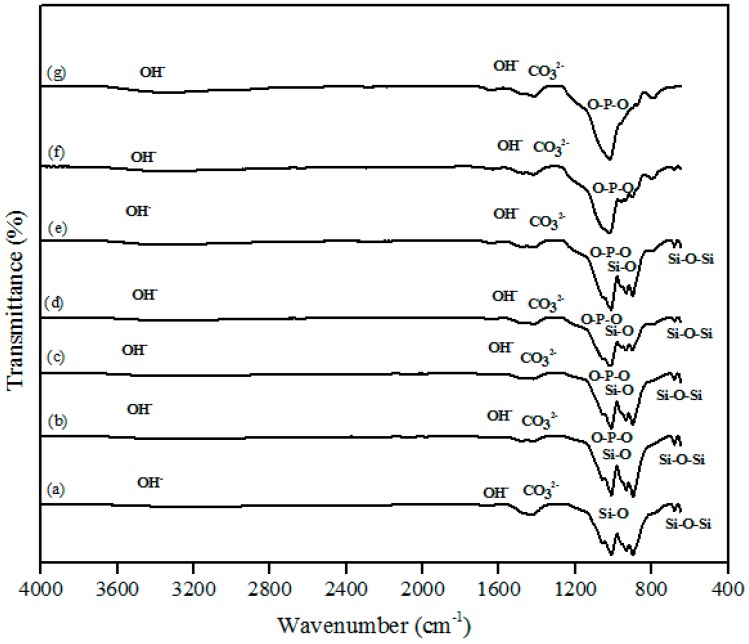
Fourier-transform infrared spectroscopy (FTIR) spectra of β-wollastonite (**a**) control sample and after soaking in the simulated body fluid (SBF) for (**b**) one; (**c**) three; (**d**) five; (**e**) seven; (**f**) 14; and (**g**) 21 days.

The FTIR spectra of the β-wollastonite samples before and after soaking in simulated body fluid (SBF) for one, three, five, seven, 14, and 21 days are shown in Figure 4. A very broad OH^−^ absorption band from 3700 to 2500 cm^−1^ and a weak water absorption band at 1650 cm^−1^ can be seen in these spectra, as similarly reported by Liu et al. [29] for all samples. The band absorption characteristic of the control sample of β-wollastonite confirmed the formation of a β-wollastonite phase based on the bending bands of silicon ions (Si–O) at 1010.50 and 931.96 cm^−1^ [1,29,30]. The stretching bands for silicon ions (Si–O–Si) at 897.08 and 898.66 cm^−1^ were indicative of β-wollastonite phases. The band between 1416 and 1424 cm^−1^ was due to the carbonate CO_3_^2−^ infrared (IR) absorption for all samples [29,30].

Previous studies have reported that the beginning of the bioactive process occurred between the 1010.50 and 931.96 cm^−1^ bands, which is shown by the existence of non-bridging oxygen stretching modes [1,29]. These bands control the rate of silicate matrix formation for the creation of the Si–OH group at the surface of the β-wollastonite. The same reaction was also detected in the sample soaked for one day, as indicated by the reduced intensity of the Si ions. This condition confirms that Si ions are required to develop the Si–OH group, which encourages the formation of apatite from day 1 until day 7 [1]. For the samples soaked for 14 and 21 days, only the bending band of P-O was observed at 1024.36 cm^−1^, which often occurs in stoichiometric apatite [31]. The existence of carbonate ions in the 1416 to 1424 cm^−1^ band may possibly be due to the carbon dioxide environment employed during the sintering process of the RHA and calcined limestone. This observation was also made by Rashita et al. in a study using silica sand and limestone [1].

**Figure 5:**
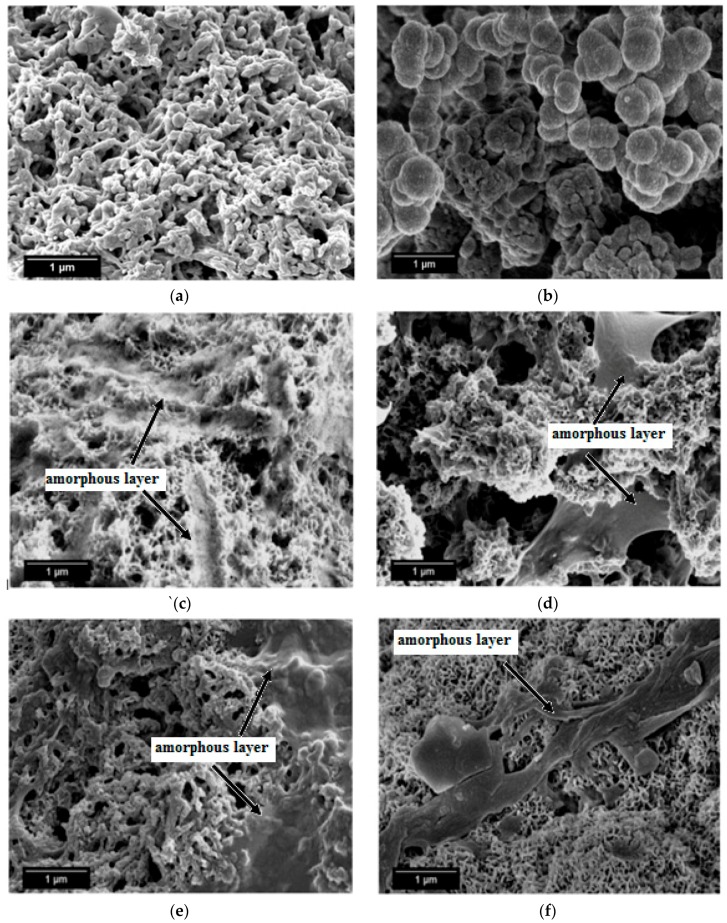

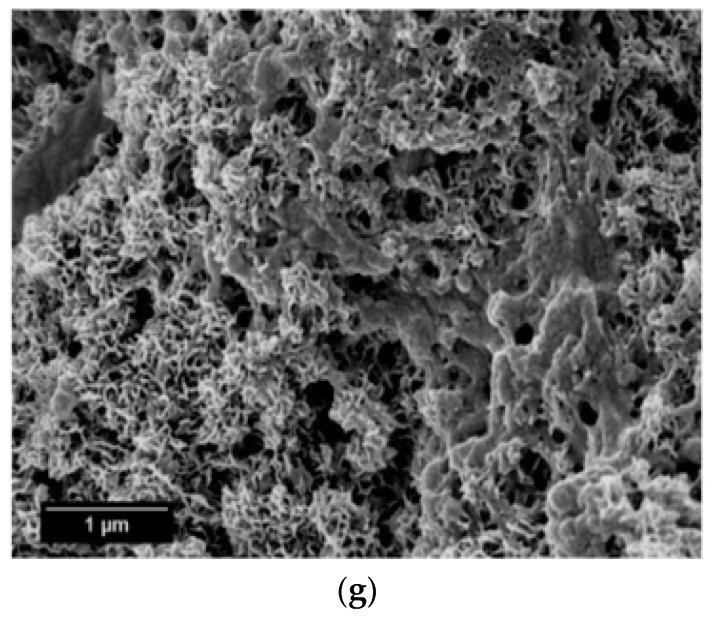
SEM micrographs of β-wollastonite (**a**) control sample and after soaking in the SBF for (**b**) one; (**c**) three; (**d**) five; (**e**) seven; (**f**) 14; and (**g**) 21 days.

SEM micrographs of the β-wollastonite samples after soaking in SBF are shown in Figure 5. The unsoaked β-wollastonite sample show a coral-like and porous structure (Figure 5a). The changes in the crystal structures of the β-wollastonite samples that were soaked for longer periods were confirmed by the mechanism of surface-directed mineralization of calcium phosphate, as introduced by Colfen [32]. When a β-wollastonite sample is soaked in SBF, the first stage of the mechanism began by producing ions such as Ca^2+^, OH^−^, H^+^, and HPO_4_^2−^. After a day of soaking, the pre-nucleation aggregates of the arrangement are in equilibrium with the ions in SBF solution, as seen in Figure 5b. After three days of soaking, this mechanism is complete. While soaking, the pre-nucleation aggregates began to form near the surface of β-wollastonite, with loose aggregates still in the solution, which occurred in stage 2 (Figure 5b). In stage 3, further aggregation occurred, causing a densification near the surface of the β-wollastonite. The nucleation of amorphous spherical particles at the surface of the β-wollastonite began to occur in stage 4. These particles appeared as a tiny layer of amorphous calcium phosphate (ACP) on the β-wollastonite (Figure 5c). Stage 5 is the last stage in the mineralization of calcium phosphate, in which crystallization occurred in the region of the amorphous particles, directed by the surface, as shown in Figure 5d. Figure 5e shows that the amorphous calcium phosphate layer became thicker and covered the surface of the β-wollastonite sample that was soaked for seven days. The amorphous calcium phosphate (ACP) layer disappeared slightly in the sample soaked for 14 days (Figure 5f), yet it appeared to be thicker again in the sample soaked for 21 days (Figure 5g). The loss of the ACP layer on the β-wollastonite surface may be caused by the factor of the Ca/P ratio, which was 1.63 after 14 days of soaking. This value is approaching the Ca/P ratio for calcium-deficient hydroxyapatite (CDHA), which ranges between 1.5 and 1.67 [33].

The energy dispersive X-ray analysis (EDX) results show that the chemical composition of the material deposited on the β-wollastonite surface consisted of Ca and P, which suggests that this material is a type of apatite. Table 3 shows the EDX analyses of the surfaces of the β-wollastonite samples that have been soaking in SBF for one, three, five, seven, 14, and 21 days. As for the control sample, the presence of a lower P peak initiates the formation of apatite when the sample is soaked in SBF solution (Table 3). The microstructure of the control sample changed to a grape-cluster shape due to the surface-directed mineralization of calcium phosphate after one day of soaking (Figure 5b). After three days, the coral-like structures had reappeared with a coated layer of amorphous spherical particles (Figure 5c). It is obvious that the Ca and P concentrations increased rapidly within the first three days of soaking and then continued to increase in a slower reaction for up to 21 days.

After soaking in SBF for different periods, the calculated Ca/P ratios for these samples were in the range of 1.2 to 2.2, as listed in Table 3. This result shows that the apatite deposited on the β-wollastonite sample consisted of ACP [33,34]. The SEM image of the microstructure of the one-day sample shows that it has a grape-cluster shape, similar to that reported by Cölfen [32]. However, the Ca/P ratio of the 14 day sample was 1.63, which is within the range of the ratio for CDHA, which is of 1.5 to 1.67 [33]. The Ca/P ratio (Table 3) increased from 1.63 to over 1.9 during the 14 to 21 days of soaking in SBF because the changing of the SBF solution will lead to the dissolution of calcium from the β-wollastonite sample in SBF solution, thus the Ca/P ratio situation cannot be controlled during a bioactivity test. Therefore, the obtained Ca/P ratio is inconsistent depending on the rate of Ca and P dissolved in the SBF solution.

The bioactivity process of wollastonite is triggered by the release of Ca and Si ions from the β-wollastonite sample when immersed in SBF. The dissolution of Ca and Si is incongruent, with Ca released preferentially relative to Si ions, thus leading to a leached layer rich in silanol (≡Si–OH). The formation of ACP and CDHA may be closely related to a number of Si-OH groups that were present on the surface of the β-wollastonite [1,27,35]. The bioactive process of the β-wollastonite material developed in this study has observed the principles of bioactive mechanism listed in the previous studies by Magallanes et al. [4] and Hench [36]. It followed one of the two principles in the bioactive process, as described below [4]. When in contact with the SBF, the β-wollastonite (CaSiO_3_) phase begins to respond with an ionic exchange of Ca^2+^ and silicate anions from the β-wollastonite network with H^+^ and OH^−^ from the SBF through to the subsequent reactions:

CaSiO_3(s)_ + H^+^_(aq.)_ → HSiO_3_^−^_(aq.)_ + Ca^2^^+^_(aq.)_ (1)

CaSiO_3(s)_ + OH^−^_(aq.)_ → Ca^2^^+^_(aq.)_ + SiO_3_(OH)_3_^−^_(aq.)_ (2)

CaSiO_3(s)_ + H_2_O_(aq.)_ → Ca^2^^+^_(aq.)_ + HSiO_3_^−^_(aq.)_ + OH^−^_(aq.)_ (3)

CaSiO_3(s)_ + H_2_O_(aq.)_ → Ca^2^^+^_(aq.)_ + SiO_4_^4^^−^_(aq.)_ + 2H^+^_(aq.)_ (4)

The silicate anions can be formed according to reactions (1) to (4). Reaction (1) has the lowest Gibbs free energy and is, therefore, the most capable [4]. According to the values of constant equilibrium, the reactions will occur in the order of (1) > (2) > (3) > (4), with HSiO_3_^−^ and Ca^2+^ being the major ions. As a result of the abundance of calcium and phosphorous ions from the β-wollastonite, many Si–OH groups can be found on the surfaces of the β-wollastonite, which formed the amorphous silica rich phase. These silanol groups are capable of initiating the heterogeneous nucleation of apatite from β-wollastonite. When apatite nuclei are formed on the surface of the β-wollastonite layer, they can quickly grow by absorbing calcium and phosphate ions from the local SBF solution [37].

**Figure 6:**
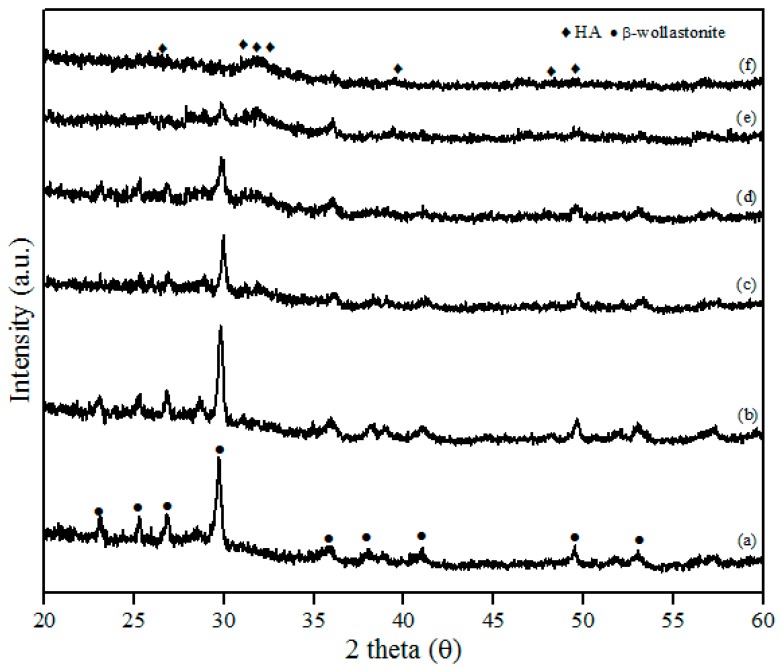
XRD patterns after soaking in the SBF for (**a**) one; (**b**) three; (**c**) five; (**d**) seven; (**e**) 14; and (**f**) 21 days.

The XRD patterns of β-wollastonite before and after soaking in SBF solution are shown in Figure 6. These results indicate that the crystallinity of β-wollastonite decreased with increasing soaking period. The ACP layer was detected in the day 3 sample and almost covered the entire surface of the β-wollastonite, as shown in Figure 5c. A broad peak around 30.0° to 35.0 degrees in Figure 6c–e refers to an ACP structure [33]. This circumstance was confirmed by the decreasing peak of β-wollastonite at 30.0 degree, based on the XRD pattern (Figure 6a–f). Hydroxyapatite peaks (ICDD number 72-1243) start to form from the converted, unstable ACP structure. Referring to Figure 6f, no β-wollastonite or ACP structure peak was observed. The HA peak was detected within 21 days of soaking. The XRD pattern shows that β-wollastonite changes from a crystalline to an amorphous structure during the soaking period (Figure 6a–f).

**Figure 7:**
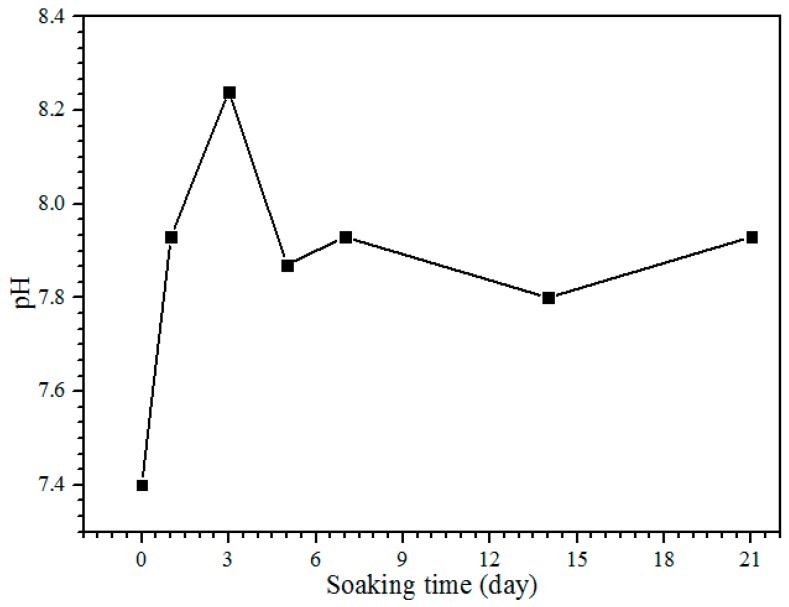
The pH of the SBF solution after each day of the soaking.

The pH of the SBF solution during the bioactivity test from day 1 until 21 is shown in Figure 7. The control pH value of the SBF solution before soaking is 7.4, with the pH and ion concentrations nearly equal to those of human blood plasma [38]. The pH solution is increased to 7.93 after one day of soaking. After day 3, the pH of the solution increases to 8.24, and it is the highest pH value obtained during the soaking process. On days 5 to 7 of soaking, the pH decreases to 7.87 but increases slightly to 7.93. The pH of the solution after days 14 and 21 is decreased to 7.80 and 7.73, respectively. The reason for the diminishing pH can be clarified because of the precipitation of calcium phosphates and carbonates in the SBF solution. The substitution of carbonate and phosphate ions would change the equilibrium of HCO^3−^ → CO_3_^2−^ + H^+^ and HPO_4_^2−^ → PO_4_^3−^ + H^+^ in the solution. This will increase and decrease the pH value [19]. The pH of the SBF solution after the bioactivity test between 5.0 and 12 is for the formation of amorphous calcium phosphate (ACP), and the pH from 6.5 to 9.5 is for calcium deficient hydroxyapatite (CDHA) formation [33]. Hence, the pH obtained in this study between 7.73 and 8.24 is subjected to the calcium deficient hydroxyapatite (CDHA) and was proven by the XRD results; the HA peak existed at day 5 of soaking (Figure 6c).

### 2.3. Degradation Study of β-Wollastonite

**Figure 8:**
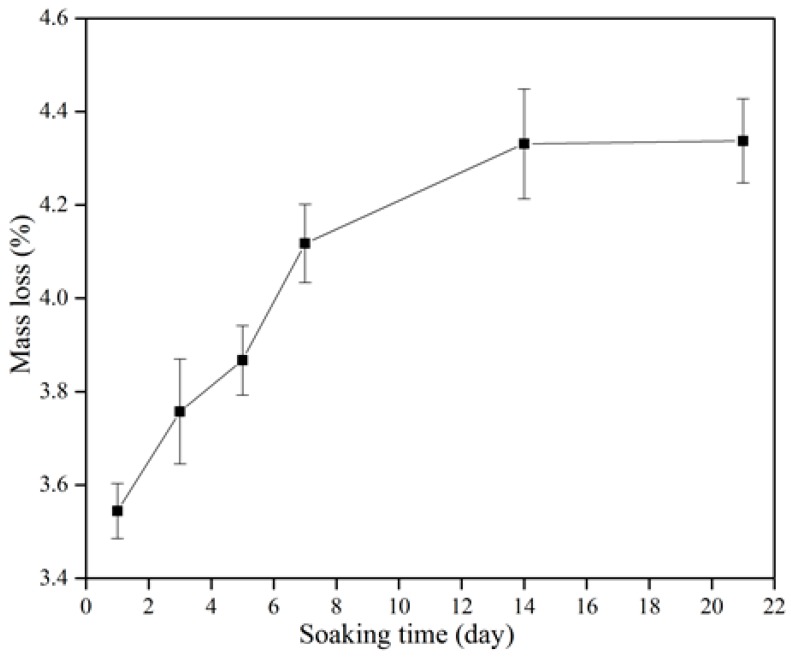
Degradation characteristics of the β-wollastonite sample.

Figure 8 shows the degradation of the β-wollastonite in the SBF. The mass loss increased incrementally as a function of soaking time. The degradability value of the β-wollastonite sample reached 3.54% after one day of soaking. As the days of soaking increased, the degradation of mass slowly increased to 3.76% at day 3, 3.87% at day 5, 4.12% at day 7, 4.33% at day 14, and finally up to 4.38% by day 21. The increasing weight loss might have been caused by the dissolution of β-wollastonite powder when it released alkaline ions while soaking in the SBF solution, as similarly reported by Li and Chang [37].

The degradation rate of β-wollastonite was also influenced by the crystallinity, sintering period, microstructure, and porosity [39,40]. In this study, the main factor for the increasing degradation rate is the porosity of the material itself. Based on the SEM images (Figure 5a), the β-wollastonite samples were very porous and had acted as the main factor in the degradation process. This was also proved by Zhang et al. [40], who stated that the degradation rate of the sintered bioceramics increased with the porosity increment due to the larger specific surface area. The samples would dissolve easily due to their increased surface area, which allowed the diffusion of dissolution to occur. Therefore, the degradation of β-wollastonite is determined by the dissolution rate, depending on the porosity and microstructure.

## 3. Materials and Methods

Limestone (CaCO_3_) powder was procured from Imerys Minerals Malaysia Sdn. Bhd., Perak, Malaysia and the rice husks were collected from Ghee Song Hong Rice Mill in Penang, Malaysia. Calcium oxide or calcined limestone powder was obtained through the process of calcining limestone at 1100 °C for 5 h. Rice husk ash (RHA) was obtained via a combustion process at 950 °C for one hour. These precursors were used as they were during the experiments.

The β-wollastonite powders were synthesized using a green synthesizing method similar to that in a previously published study [41], and no significant controls need to be considered. The chemical elemental analyses of the raw materials were conducted using X-ray Fluorescence (XRF, S8 Tiger, Bruker, Karlsruhe, Germany). The particle size analyses and the density of the precursors and sintered powders were measured using a particle sizer Microtrac-X100 and aPycnometer AccuPyc 1340. The sintered powders were characterized using X-ray diffraction (XRD, D8 Advance, Bruker, Karlsruhe, Germany). In addition, the heavy element content was completed by Inductively Coupled Plasma Atomic Emission Spectroscopy analysis (ICP-AES, Optima 4300 DV, Perkin Elmer, Waltham, MA, USA). The rice husk ash, calcined limestone, and β-wollastonite weighted as much as 0.4 g and were dissolved in 250 ml nitric acid with 1 M concentration. A total of 15 ml of the solution for each sample was inserted into the induction plasma argon core. When the temperature reached 8000 °C, we measured elements such as arsenic (As), cadmium (Cd), lead (Pb), and mercury (Hg). Fourier transform infrared spectroscopy (FTIR-ATR, Perkin Elmer, Waltham, MA, USA) was also carried out on the yield powder containing KBr pellets. All spectra were recorded in absorption mode at 2 cm^−1^ intervals and in the wavelength range of 4000 to 650 cm^−1^. The morphology and elemental analyses of β-wollastonite powder were observed using a field-emission scanning electron microscope (FESEM, Merlin Compact, Zeiss, Jena, Germany), coupled with energy-dispersive X-ray spectroscopy (EDS, IncaEnergy, Oxford Instrument, Oxfordshire, United Kingdom).

Before the cell viability test was carried out, disc shapes with radii of 10 mm and heights of 1 mm of the RHA, calcined limestone, and β-wollastonite samples were steam sterilized (120 °C, 20 min), referring to the method used by Swain et al. [42]. The cell viability test was evaluated by using PrestoBlue™ reagent as per standard protocol to assess cell viability. Human osteoblasts (differentiated from human mesenchymal stem cells) were seeded in the culture medium (Dulbecco’s modified eagle’s medium/nutrient mixture F-12 ham (FD); HEPES buffer solution 1 M in H_2_O from ThermoFisher, Scientific, Waltham, MA, USA; GlutaMAX™ (100X) from ThermoFisher, Scientific, Waltham, MA, USA; antibiotics and antimitotics; and fetal bovine serum (FBS); dexamethasone; beta-glycerophosphate; and ascorbate phosphate), which was cultured at a density of 200 k cells per 48-well culture plate and cultured in an incubator at 37 °C for one and three days to allow cell adhesion. The optical density of the solution was measured at 560 nm by an ELISA reader, and then the cell distribution and cell growth were calculated. The cell viability for each sample was performed with n = 3, and the difference in cell number and cell growth rate across was statistically analysed materials using the Kruskal-Wallis Test. Cells seeded on plastic culture dish surface were used as a control.

To study its bioactivity, 1 g of β-wollastonite powder was placed in the Teflon mould and pressed, using a glass rod, into a cylindrical shape of 12 mm high and 6 mm in diameter. SBF was prepared according to the method described by Kokubo and Takadama [38], with an ion concentration nearly equal to that of human blood plasma. Then, the duplicates of the β-wollastonite samples were soaked in SBF at pH 7.4 and a temperature of 36.5 °C for one, three, five, seven, 14, and 21 days respectively. The SBF solution was changed every three days. After the soaking period, the β-wollastonite samples were washed in acetone for 2 h, followed by three rinses with deionized water to remove buffer salts. Then they were dried in an incubator for 24 h. Next, the β-wollastonite samples were characterized using Fourier transforms infrared (FTIR) spectroscopy (FTIR-ATR, Perkin Elmer, Waltham, MA, USA) and FESEM (FESEM, Merlin Compact, Zeiss, Jena, Germany) coupled with EDS. The Ca/P ratios for the β-wollastonite samples were calculated using the atomic percentages of Ca and P, which were obtained from the EDS (EDS, IncaEnergy, Oxford Instrument, Oxfordshire, UK) chemical analysis.

The degradability of the duplicate β-wollastonite was determined from its percentage weight loss after soaking in SBF, as mentioned before. The initial weight (mi) of the cylindrical samples (12 by 6 mm) was measured before soaking in the SBF. The dried samples were measured (md), and, finally, the weight loss of the β-wollastonite was measured and calculated using the following formula (5):

Weight loss (%) = 100 × {(mi – md)/(mi)}(5)

## 4. Conclusions

β-wollastonite was successfully produced from rice husk ash and calcined limestone via green synthesizing using autoclaving with good bioactivity properties. The morphology of the β-wollastonite samples changed during the soaking process, with significant Ca and P ion contents, which helped the formation of amorphous calcium phosphate and calcium-deficient hydroxyapatite on the surface of the sample. The β-wollastonite sample also went through changing phases from crystalline to amorphous. Both the starting materials and the β-wollastonite showed biocompatibility properties with the cell. Since the β-wollastonite sample is porous, it revealed significant degradation during the soaking process. The degradation rate of the β-wollastonite increased with the increasing length of the soaking period. Hence, analyses such as mechanical testing need to be carried out to determine the mechanical properties of the synthesized β-wollastonite in order to support it as an implantable material.

## Figures and Tables

**Table 1:** Composition of the raw materials.

Composition	Calcined Limestone (wt %)	Rice Husk Ash (RHA) (wt %)
CaO	97.22	0.57
MgO	2.38	1.24
P_2_O_5_	-	3.36
SiO_2_	-	89.50
Al_2_O_3_	-	0.58
K_2_O	-	3.61
Others	0.4	1.14

**Table 2:** Trace heavy element of rise husk ash (RHA), β-wollastonite.

Sample	Heavy Element Content (ppm)			
	As	Cd	Pb	Hg
ASTM F 1538-03	3	5	30	5
RHA	0.022	0.003	0.007	0
Calcined limestone	0.017	0	0.058	0
β-wollastonite	0.023	0.003	0.002	0

**Table 3:** Surface composition of the β-wollastonite samples before and after soaking in SBF solution.

Soaking Period (day)	Surface Composition of β-Wollastonite (at. %)		Molar Ratio Ca/P
	Ca	P	
0	15.36	2.57	-
1	7.47	6.29	1.19
3	13.53	7.25	1.87
5	18.02	8.05	2.24
7	18.78	9.31	2.02
14	18.27	11.21	1.63
21	22.32	11.64	1.92
